# Metabolic network visualization eliminating node redundance and preserving metabolic pathways

**DOI:** 10.1186/1752-0509-1-29

**Published:** 2007-07-03

**Authors:** Romain Bourqui, Ludovic Cottret, Vincent Lacroix, David Auber, Patrick Mary, Marie-France Sagot, Fabien Jourdan

**Affiliations:** 1 LaBRI, Université Bordeaux I, 351 Cours de la libération, 33405 Talence CEDEX, France; 2 BAOBAB Team, Inria Rhône-Alpes, Projet HELIX, Université de Lyon ; université Lyon 1 ; CNRS ; UMR 5558, Laboratoire de Biométrie et Biologie Evolutive, 43 boulevard du 11 novembre 1918, Villeurbanne F-69622, France; 3 UMR1089 Xénobiotiques INRA-ENVT, 180 chemin de Tournefeuille – St-Martin-du-Touch, BP 3, 31931 Toulouse CEDEX, France

## Abstract

**Background:**

The tools that are available to draw and to manipulate the representations of metabolism are usually restricted to metabolic pathways. This limitation becomes problematic when studying processes that span several pathways. The various attempts that have been made to draw genome-scale metabolic networks are confronted with two shortcomings: 1- they do not use contextual information which leads to dense, hard to interpret drawings, 2- they impose to fit to very constrained standards, which implies, in particular, duplicating nodes making topological analysis considerably more difficult.

**Results:**

We propose a method, called MetaViz, which enables to draw a genome-scale metabolic network and that also takes into account its structuration into pathways. This method consists in two steps: a clustering step which addresses the pathway overlapping problem and a drawing step which consists in drawing the clustered graph and each cluster.

**Conclusion:**

The method we propose is original and addresses new drawing issues arising from the no-duplication constraint. We do not propose a single drawing but rather several alternative ways of presenting metabolism depending on the pathway on which one wishes to focus. We believe that this provides a valuable tool to explore the pathway structure of metabolism.

## Background

### Metabolism visualization for systems biology studies

The scale of metabolic studies varies according to the data and to the biological questions. For instance, toxicologists often follow the degradation of a given molecule; in that case they focus only on a very small number of reactions. At a larger scale, biologists studying glycolysis will focus on this particular metabolic pathway. Most of the work on metabolism visualization has been done at this level of detail [1-12]. However, in order to investigate an organism's metabolic response to stress, it is relevant to study all the pathways simultaneously. For instance, this will be useful for treating the results of high throughput experiments such as transcriptomic data where relevant gene products are identified in many pathways. Visualization is a suitable and obvious solution to achieve this kind of study, for instance by representing all the metabolic pathways in one drawing and by coloring relevant enzymes and metabolites [13-15]. In [16], the authors use this approach to analyze simultaneously transcriptomic and metabolomic data (they used Biocyc *omics viewer *[14]). Based on this representation, they managed to identify at once perturbations in the Calvin cycle, glycolysis and TCA cycle. Such kinds of studies emphasize the necessity to develop methods that allow to visualize the entire metabolic network in a single drawing.

Highlighting pathways according to experimental data provides some clues on metabolic processes. However, to integrate these conclusions in a systems biology approach, it is necessary to understand how these pathways are linked and how processes span over them.

The issue of analyzing biological processes spanning several metabolic pathways appears in many contexts. As we already mentioned, it appears when analyzing metabolomic or transcriptomic experiments, which are generally not pathway-focused. This issue also arises for topological analyses based on motif detection [17]. A motif (defined as a set of reaction types) may occur in different parts of the network (which illustrates the need to visualize the whole network in a single picture), and each occurrence may be composed of reactions belonging to different pathways (which examplifies the need to explicitly visualize the links between the pathways).

Therefore, pathway visualization is not suitable for such tasks but neither is network visualization without pathway information. Indeed, to be useful for mapping experiments, it is necessary to represent the entire network structure while keeping the contextual information provided by its division into metabolic pathways. Note that this is one of the requirements for biological network visualization proposed in [18]. Recently, in addition to the studies that use the network as a background, great efforts have been devoted to the analysis of the topological properties of metabolic networks [19,20]. Indeed topology could, for instance, give clues on the evolution of the organisms they are related to. More generally, topological features like shortest path, connectivity, node degrees and node/edge metrics have become common investigation tools. To visually retrieve topological information, it is necessary that the drawing provides a faithful image of the network structure. This is a challenging problem which has not been addressed by current metabolic network visualization tools [13,14] which choose to allow node duplication and therefore do not face this issue.

In the case where nodes are not duplicated, pathways which share reactions and compounds cannot all be drawn equally well (a well-drawn pathway being a pathway having all its nodes drawn next to each other). Therefore, choices have to be made on which pathways will be drawn well in priority. We propose both an automatic way of making this choice and possibilities for the user to define his own priorities. This last option adds an interesting feature to the tool: depending on the choices made, the backbone of metabolism (the set of well-drawn pathways) can be adjusted to the pathways one is interested in. This backbone can either include the glycolysis and the TCA cycle as it is traditionnally the case in most drawings or, alternatively, it can include pathways that share compounds or reactions with glycolysis and the TCA cycle and which would, if not chosen, be drawn in the background. Playing around with this option enables to get a grip on the interdependence of the pathways.

The aim of this paper is to propose an algorithm to draw the entire metabolic network. The produced representation will have to follow textbook drawing conventions (see the following section), display information on the metabolic pathways and keep the topology of the network by avoiding node duplication.

### Metabolic network drawing and visualization

#### Drawing metabolic pathways

A metabolic pathway (also called a metabolic map) is a subnetwork of the metabolic network. The decomposition of the entire network into metabolic pathways is generally done according to biological functions: molecule degradation (catabolism), molecule synthesis (anabolism) or energy transfer [21]. Until recently, these pathways have been manually drawn, for instance for teaching purposes, or to exchange results [22,23]. Then, numerical versions of these manual drawings were proposed and used on web servers such as KEGG [3,24].

In the last few years, automatic drawing algorithms have been designed, mainly for two reasons. First the number of organisms for which a metabolic network is described is increasing quickly. Indeed, *in silico *methods have been designed to reconstruct metabolic pathways from annotated genomes [25] which are more and more numerous. Second, these putative networks follow a regular curating process implying many changes in their structures. In this section, we describe the algorithms that have been proposed for drawing metabolic pathways since they could be extended to the entire network.

Because biologists are used to textbook representations, most of the automatic methods consist in following the drawing habits of these representations [22]. Even if there is no standard for these conventions, it is possible to identify the most commonly used ones. Some of the aesthetic criteria are also used in graph drawing [26-28]: lowering the number of edge crossings and lowering the number of bends on edges. Moreover, the biological nature of pathways implies some conventions. The notion of reaction cascade is central since generally metabolic pathways describe the transformation of input metabolites into output ones. Most automatic drawing algorithms have been designed to emphasize this structure. The algorithm proposed in [5] and implemented in Biominer uses a hierarchical drawing algorithm which embeds nodes on regular horizontal layers [29]. Others propose adapted versions of classical hierarchical drawing algorithms, like in [6] (implemented in BIOPATH [30]) or in [9] (implemented in Wilmascope).

**Figure 1:**
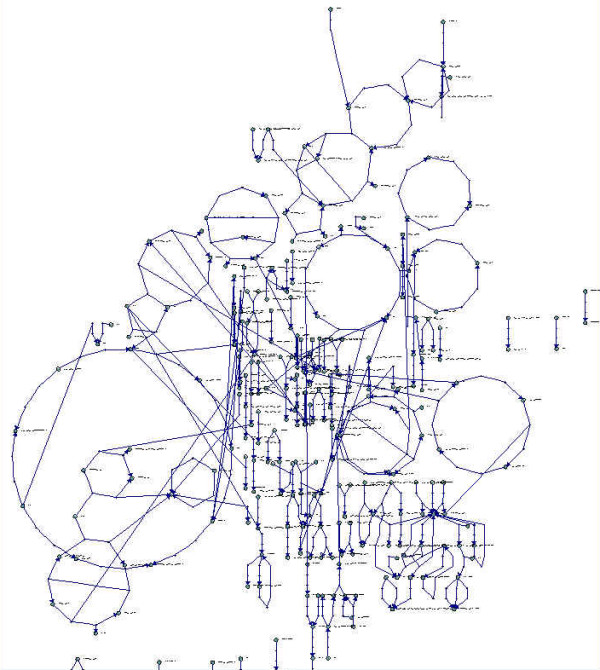
*Mus musculus *metabolic network drawn using SimWiz software implementing [10] algorithm. This network appears to be smaller than the one of *E. coli*. This is simply due to the fact that our knowledge of mouse metabolism is very partial.

However, these algorithms do not emphasize cyclic patterns which are also relevant (see for instance the TCA cycle). Thus, other methods were designed to take into account these two configurations. The first one was proposed in [4] where the authors introduce a compound graph layout algorithm, that is, they first detect cycles then treat them as metanodes creating a Directed Acyclic Graph (DAG) and applying a hierarchical drawing algorithm on this DAG. In [10], the authors refine the approach by detecting nodes shared by two cycles thus providing two cyclic representations instead of one. Finally, [11] proposed the same kind of approach for signaling pathways, adding the ability to manually constrain the drawing. However, all these algorithms were initially designed to draw pathways and are not well adapted to draw networks. For instance, we tried to use the software SimWiz which implements the algorithm proposed in [4] to draw the metabolic network of *Escherichia coli *but the program failed because the network was too large. We were nevertheless able to draw the metabolic network of *Mus musculus*, which is smaller. The result is shown in figure 1. In this case, the main problem is due to the cycle detection which is applied on the whole network thus highlighting cycles that span over different pathways.

#### Scaling to the whole metabolic network

In the Graph Drawing community, efficient drawing algorithms have been designed to draw large networks. Among them, force-based layouts [31,32] are commonly used. Such layouts mimic physical systems, that is, nodes are considered as masses (or particles) and edges behave as springs (or magnetic forces). This system evolves from a random embedding to one corresponding to an equilibrium, providing a suitable layout. These algorithms generate quite good drawings since they generally emphasize dense subgraphs and spread low degree nodes on the screen space. They are used in Cytoscape [33] or in the online SBML viewer [34] for instance. However, as mentioned in [18], such drawings are not satisfying for biologists. The first reason is that they do not follow textbook drawing conventions, and the second is that they emphasize topological clusters which generally do not correspond to a metabolic pathway decomposition. To overcome this last problem, force-based methods could be used in a compound graph layout as it is done in [8] (implemented in PatikaWeb [12]). However, this tool is not dedicated to metabolic pathway visualization and thus does not follow all textbook drawing conventions.

The two main efforts for automatically drawing metabolic networks while keeping metabolic pathway information and respecting drawing conventions are: Reactome [13] and the Pathway Tools cellular overview diagram [14]. As it was mentioned before, in both tools nodes are duplicated thus the only drawing problem is to embed metabolic maps. Both achieve it by grouping maps according to their common functions. The latter assumes that a hierarchy on the pathways is given as input to the algorithm and is then used to display pathways close to each other when they are close to each other in the hierarchy. This functionality is not included in the current implementation of our algorithm. Nevertheless, it is still possible to circumvent this problem by redefining coarse-grained pathways (corresponding to groups of pathways of common functions) in the input data.

In the following sections, we first describe our metabolic network drawing algorithm. Then we discuss our approach and compare it to other published methods using the metabolic network of *Esherichia coli *(*E. coli*) as benchmark.

## Implementation

### Using a mixed bipartite graph to model metabolic networks

A graph provides an intuitive way of organizing large amounts of relational data. The general definition of a graph *G *= (*V*, *E*) is simple. It consists of a set *V *of *n *vertices (|*V*| = *n*) and a set *E *of *m *edges, each of which corresponds to a pair-wise relationship between two of the nodes (*E *⊆ *V *× *V*). Modeling the metabolic network consists in choosing which biological objects are associated to nodes and edges. It is necessary to do this model description before introducing the graph drawing algorithm, since it will constrain the representation. For instance, a model may imply that some nodes have a high degree, thus complicating a planarization process.

#### Bipartite graph

**Figure 2:**
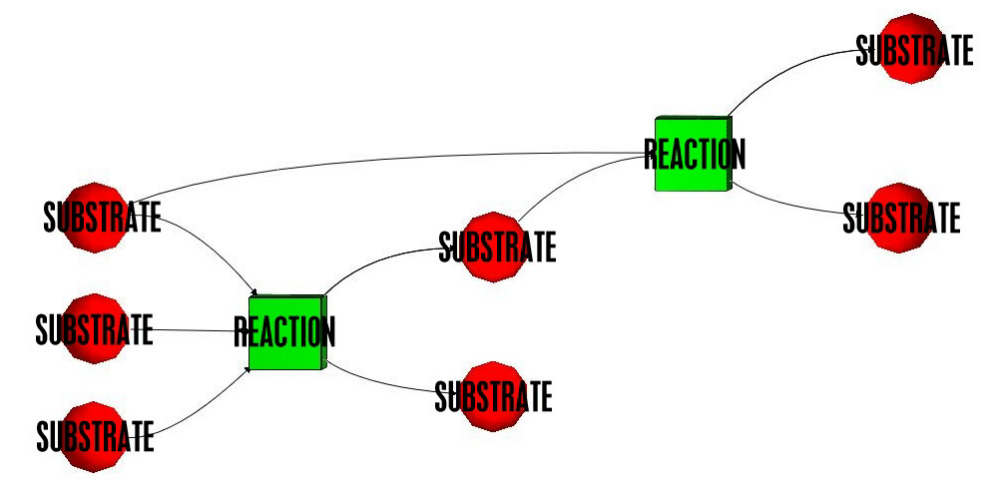
Bipartite graph describing two biochemical reactions.

A metabolic network is a set of biochemical reactions (*i.e*. reactions that convert one or more compounds into one or more other compounds). Different models could be used (for a detailed discussion, see [35]). Here, we consider that there are two kinds of nodes: reactions and substrates (see Figure 2) and that there is an edge between a reaction and a substrate if the substrate is consumed or produced by the reaction. The discussion of this choice is out of the scope of this paper, but the main motivation is due to the use of this model in many textbook drawings. This graph is generally called a *bipartite *graph since its set of nodes can be split into two subsets where the elements are not linked (no link between reactions and no link between substrates). Thus the set of vertices can be split into two subsets *R *= {*v *∈ *V *|*v *is a reaction} and *S *= {*v *∈ *V *|*v *is a substrate}, and *V *= *R *⊕ *S *and *E *⊆ {(*u*, *v*)|*u *∈ *R*, *v *∈ *S*} = *R *× *S*.

#### Mixed graph

Metabolic reaction can be either reversible (*i.e*. it can occur in both directions) or irreversible (*i.e*. it can occur in only one direction). This orientation is defined according to the physiological properties of a reaction. SBML descriptions of reactions provide this kind of information. In order to model such a biological phenomenon, we use a *mixed *graph. In a mixed graph, the set *E *of edges is splitted in two subsets *A *and *E'*, where *A *is the set of *arcs *(*i.e*. oriented edges), *E' *is a set of non-oriented edges and *E *= *A *⊕ *E'*.

Thus, for modeling the whole network, we use a *mixed bipartite *graph *G *= (*R*, *S*, *A*, *E'*).

#### Graph hierarchy

A metabolic pathway is a subnetwork of the metabolic network. Here, it corresponds to a graph *G*_*p *_= (*V*_*p*_, *E*_*p*_) where *V*_*p *_⊂ *V *and *E*_*p *_= {(*u*, *v*) ∈ *E*|*u *∈ *V*_*p *_and *v *∈ *V*_*p*_} ⊂ *E *(*i.e*. *E*_*p *_is the set of edges and arcs induced by *V*_*p *_on *E*). For a given metabolic network *G*, we note *P*_*G *_= {*G*_*i*_| 1 ≤ *i ≤ n*_*p*_} its *n*_*p *_metabolic pathways. One can notice that for each *G*_*i*_, *V*_*i *_and *E*_*i *_can be decomposed in four subsets *R*_*i*_, *S*_*i*_, *A*_*i *_and E′i
 MathType@MTEF@5@5@+=feaafiart1ev1aaatCvAUfKttLearuWrP9MDH5MBPbIqV92AaeXatLxBI9gBaebbnrfifHhDYfgasaacH8akY=wiFfYdH8Gipec8Eeeu0xXdbba9frFj0=OqFfea0dXdd9vqai=hGuQ8kuc9pgc9s8qqaq=dirpe0xb9q8qiLsFr0=vr0=vr0dc8meaabaqaciaacaGaaeqabaqabeGadaaakeaacuWGfbqrgaqbamaaBaaaleaacqWGPbqAaeqaaaaa@2F52@ (*i.e*. *G*_*i *_is a mixed bipartite graph).

Taking pathways into account leads to the following graph hierarchy : the graph *G *representing the whole network and *n*_*p *_induced subgraphs *G*_*i *_representing its *n*_*p *_metabolic pathways.

### Drawing algorithm

The algorithm we propose has two main steps: first, a multi-scale clustering is performed creating a quotient graph (strictly speaking, the quotient graph is built by considering isolated nodes as singletons), and second, clusters and quotient graph are drawn using three drawing algorithms. In the next section, we first explain our clustering algorithm and then, we present the drawing algorithms we use.

#### Multi-scale clustering

**Figure 3:**
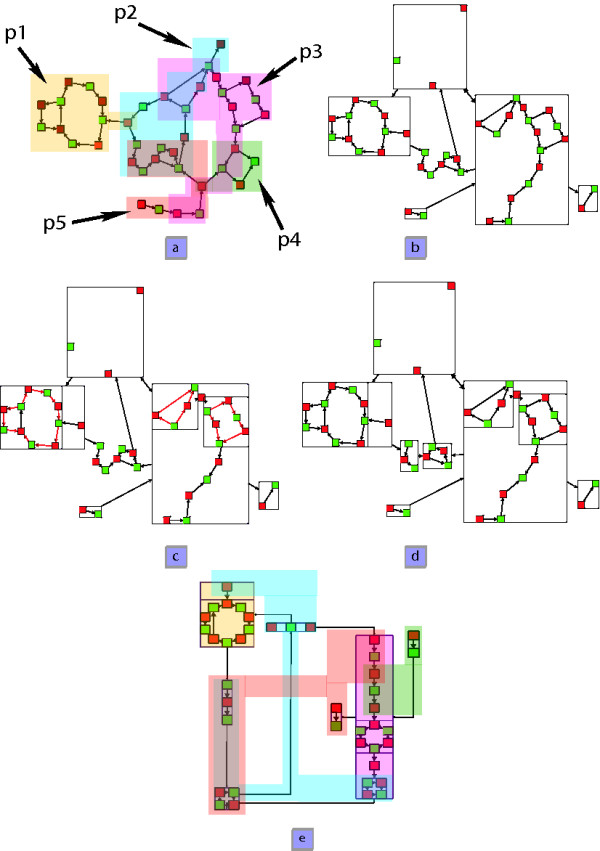
**Algorithm overview**. (a) a network where each pathway is depicted by a color (b) clustering according to metabolic pathways overlapping (c) cycles detection in metanodes (d) cycles and paths detection (e) final representation

One of the main problems is that metabolic pathways often share nodes. For instance, in Figure 3, the yellow, blue and purple regions respectively represent pathways *p*_1_, *p*_2 _and *p*_3_. One can see an overlap between *p*_1 _and *p*_2 _(one node) and between *p*_2 _and *p*_3 _(four nodes). This situation is not rare in real networks: in the *E. coli *metabolic network, 658 nodes (out of a total of 1140) are shared between several pathways, and the average number of pathways per node is more than 2.4. Since we choose not to duplicate nodes, and since vertices of a pathway have to be drawn next to each other, our algorithm has to decide whether a node is embedded next to a pathway or next to another. For example, the shared node between *p*_1 _and *p*_2 _could be drawn near *p*_1 _or near *p*_2_. This is achieved by a two-step process. The first step consists in computing an independent set of pathways (*i. e*. a set of pathways which do not share nodes) and the second one in detecting cycles and paths.

##### First pass : computation of an independent set of pathways

First of all, the algorithm searches for a subset *P*_*ind *_= {*p*_1_, ..., *p*_*ind*_}, *ind *≥ 1, *P*_*ind *_⊆ *P*_*G *_such that 1. the pathways of *P*_*ind *_are independent and 2. ∑i=1i=ind|pi|
 MathType@MTEF@5@5@+=feaafiart1ev1aaatCvAUfKttLearuWrP9MDH5MBPbIqV92AaeXatLxBI9gBaebbnrfifHhDYfgasaacH8akY=wiFfYdH8Gipec8Eeeu0xXdbba9frFj0=OqFfea0dXdd9vqai=hGuQ8kuc9pgc9s8qqaq=dirpe0xb9q8qiLsFr0=vr0=vr0dc8meaabaqaciaacaGaaeqabaqabeGadaaakeaadaaeWaqaamaaemaabaGaemiCaa3aaSbaaSqaaiabdMgaPbqabaaakiaawEa7caGLiWoaaSqaaiabdMgaPjabg2da9iabigdaXaqaaiabdMgaPjabg2da9iabdMgaPjabd6gaUjabdsgaKbqdcqGHris5aaaa@3E8D@ is maximized. For instance, in Figure 3a, {*p*_1_, *p*_3_} is the independent set that maximizes this sum among all possible independent sets of pathways ({*p*_1_}, {*p*_2_},{*p*_3_}, {*p*_4_}, {*p*_5_}, {*p*_1_, *p*_3_}, {*p*_1_, *p*_4_}, {*p*_1_, *p*_5_}, {*p*_2_, *p*_4_} and {*p*_4_, *p*_5_}).

The problem of finding a maximum independent set is known to be NP-Hard [36]. This problem can be reduced to a coloration problem (the graph is then the dependence graph, where each pathway corresponds to a node and there is an edge between two nodes when the pathways share nodes in the original graph). To find a solution, we use the Welsh and Powel heuristic [37]. Then, for each color class C, ∑pi∈C|pi|
 MathType@MTEF@5@5@+=feaafiart1ev1aaatCvAUfKttLearuWrP9MDH5MBPbIqV92AaeXatLxBI9gBaebbnrfifHhDYfgasaacH8akY=wiFfYdH8Gipec8Eeeu0xXdbba9frFj0=OqFfea0dXdd9vqai=hGuQ8kuc9pgc9s8qqaq=dirpe0xb9q8qiLsFr0=vr0=vr0dc8meaabaqaciaacaGaaeqabaqabeGadaaakeaadaaeqaqaamaaemaabaGaemiCaa3aaSbaaSqaaiabdMgaPbqabaaakiaawEa7caGLiWoaaSqaaiabdchaWnaaBaaameaacqWGPbqAaeqaaSGaeyicI4Saem4qameabeqdcqGHris5aaaa@3A3A@ is computed, and a maximum one is chosen as our independent set.

Let *P*_*Nind *_= *P*_*G*_\*P*_*ind*_. Then, for all the pathways in *P*_*Nind*_, we exclude nodes that are shared with at least one other pathway in *P*_*G*_. We denote this reduced set by P′Nind
 MathType@MTEF@5@5@+=feaafiart1ev1aaatCvAUfKttLearuWrP9MDH5MBPbIqV92AaeXatLxBI9gBaebbnrfifHhDYfgasaacH8akY=wiFfYdH8Gipec8Eeeu0xXdbba9frFj0=OqFfea0dXdd9vqai=hGuQ8kuc9pgc9s8qqaq=dirpe0xb9q8qiLsFr0=vr0=vr0dc8meaabaqaciaacaGaaeqabaqabeGadaaakeaacuWGqbaugaqbamaaBaaaleaacqWGobGtcqWGPbqAcqWGUbGBcqWGKbazaeqaaaaa@3343@.

Each element of *P*_*ind *_and P′Nind
 MathType@MTEF@5@5@+=feaafiart1ev1aaatCvAUfKttLearuWrP9MDH5MBPbIqV92AaeXatLxBI9gBaebbnrfifHhDYfgasaacH8akY=wiFfYdH8Gipec8Eeeu0xXdbba9frFj0=OqFfea0dXdd9vqai=hGuQ8kuc9pgc9s8qqaq=dirpe0xb9q8qiLsFr0=vr0=vr0dc8meaabaqaciaacaGaaeqabaqabeGadaaakeaacuWGqbaugaqbamaaBaaaleaacqWGobGtcqWGPbqAcqWGUbGBcqWGKbazaeqaaaaa@3343@ is a set of nodes. These sets define a clustering on the original graph since there is no overlapping between them. This clustering is used by replacing each subgraph induced by an element of *P*_*ind *_or P′Nind
 MathType@MTEF@5@5@+=feaafiart1ev1aaatCvAUfKttLearuWrP9MDH5MBPbIqV92AaeXatLxBI9gBaebbnrfifHhDYfgasaacH8akY=wiFfYdH8Gipec8Eeeu0xXdbba9frFj0=OqFfea0dXdd9vqai=hGuQ8kuc9pgc9s8qqaq=dirpe0xb9q8qiLsFr0=vr0=vr0dc8meaabaqaciaacaGaaeqabaqabeGadaaakeaacuWGqbaugaqbamaaBaaaleaacqWGobGtcqWGPbqAcqWGUbGBcqWGKbazaeqaaaaa@3343@ by a metanode representing it (see Figure 3b). We call this first clustered graph *G*_*clust*1_.

For all the pathways in *P*_*ind *_and in P′Nind
 MathType@MTEF@5@5@+=feaafiart1ev1aaatCvAUfKttLearuWrP9MDH5MBPbIqV92AaeXatLxBI9gBaebbnrfifHhDYfgasaacH8akY=wiFfYdH8Gipec8Eeeu0xXdbba9frFj0=OqFfea0dXdd9vqai=hGuQ8kuc9pgc9s8qqaq=dirpe0xb9q8qiLsFr0=vr0=vr0dc8meaabaqaciaacaGaaeqabaqabeGadaaakeaacuWGqbaugaqbamaaBaaaleaacqWGobGtcqWGPbqAcqWGUbGBcqWGKbazaeqaaaaa@3343@, we search for the longest independent mixed cycles (Cycles *C*_1 _and *C*_2 _are independent if *C*_1 _and *C*_2 _do not share any node). A mixed cycle is a sequence of nodes *v*_1_, *v*_2_, ..., *v*_*l*_, *l *≥ 3 such that ∀ 1 <*i *≤ *l*, (*v*_*i*-1_, *v*_*i*_) ∈ *E' *∪ *A *and (*v*_*l*_, *v*_1_) ∈ *E' *∪ *A*.

Moreover, ∀ 1 <*i *<*l*, if *v*_*i *_represents a reaction and *v*_*i*-1 _a substrate consumed in (resp. produced by) this reaction, then *v*_*i*+1 _is produced by (resp. consumed in) *v*_*i*_. This problem is also NP-Complete even if *A *= ∅ [36]. To "solve" it, we use an exact maximum length cycle algorithm and bound the computation time with a threshold. If the threshold is reached, we stop the algorithm and consider that the longest mixed cycle we have already found is a longest one. This allows to have an exact result in the best case and an approximation of a longest mixed cycle otherwise. The technique computes all mixed paths using a *mixed *breadth-first search (BFS). In Figure 3c, one can see the longest independent cycles of each element of *P*_*ind *_and P′Nind
 MathType@MTEF@5@5@+=feaafiart1ev1aaatCvAUfKttLearuWrP9MDH5MBPbIqV92AaeXatLxBI9gBaebbnrfifHhDYfgasaacH8akY=wiFfYdH8Gipec8Eeeu0xXdbba9frFj0=OqFfea0dXdd9vqai=hGuQ8kuc9pgc9s8qqaq=dirpe0xb9q8qiLsFr0=vr0=vr0dc8meaabaqaciaacaGaaeqabaqabeGadaaakeaacuWGqbaugaqbamaaBaaaleaacqWGobGtcqWGPbqAcqWGUbGBcqWGKbazaeqaaaaa@3343@ highlighted in red. These cycles are clustered into metanodes yielding a multi-scale graph called *G*_*clust*2_. For all the metabolic networks on which we tested our algorithm, the threshold was not reached (*i.e*. we found an exact solution).

##### Second pass : detection of cycles and paths

The next step of the algorithm consists in computing the longest independent mixed cycles in *G*_*clust*2_, excluding metanodes. At each iteration, we cluster a longest cycle into a metanode and exclude it for the next search. We then compute the longest mixed paths, i.e. the longest sequences of nodes of degree less or equal to two *v*_1_, *v*_2_, ..., *v*_*l*_, *l *≥ 2, where ∀1 <*i *≤ *l*, (*v*_*i*-1_, *v*_*i*_) ∈ *E' *∪ *A*.

In figure 3d, one can see the two new metanodes, the left one is a path and the other one is a cycle. The result of this clustering is the quotient graph that will be the input of the drawing algorithm.

#### Drawing algorithm

To draw the metabolic network, we use three drawing algorithms: one for the quotient graph and two for the metanodes.

##### Drawing metanodes

**Figure 4:**
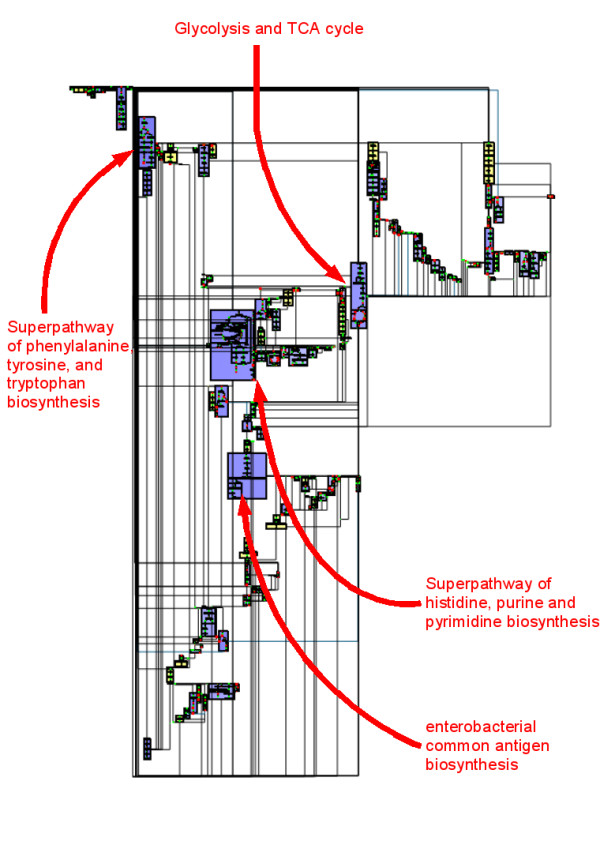
**Whole metabolic network of E. coli drawn by MetaViz**. The metanodes in purple represent metabolic pathways completely drawn. The metanodes in yellow correspond to specific structural schemes (chains or cycles) found by MetaViz.

To draw subgraphs represented by metanodes, we use a recursive drawing algorithm. This algorithm draws all the subgraphs from the most nested to the least nested. According to our clustering method, a subgraph is either a cycle or an acyclic graph. In the first case, we use a circular drawing algorithm (see figure 4); in the second case, we use the hierarchical drawing algorithm presented in [38].

##### Drawing the quotient graph

We want a drawing that optimizes the angular resolution and the number of bends to obtain a better visibility. The Mixed-Model algorithm of C. Gutwenger and P. Mutzel [39] is a trade-off between all these aesthetic criteria. Moreover, drawings produced by this algorithm are similar to manually drawn metabolic networks.

To use the Mixed-Model algorithm, we need to make modifications on the quotient graph. Indeed, it can only be applied to planar graphs; therefore, we have to planarize (*i.e*. make it planar) the quotient graph. This problem is well-known and is NP-Hard [40]. Many techniques exist that do it either by augmentation or by deletion of edges (or nodes). For a survey on this topic, one can refer to [41]. The drawback of an augmentation based technique is that it may add up to |*V*|^4 ^nodes, thus the drawing becomes difficult to understand. That is why we use our own heuristic: vertices of higher degree are removed one by one until the graph becomes planar. All removed nodes are then re-inserted. Removed edges are re-added one by one as long as the graph is planar.

The re-insertion of edges for each node is done with no prior order, using a greedy approach. The edges that have been removed and not re-inserted during the planarization step will be re-inserted after the planar subgraph is drawn.

The obtained planar subgraph of the quotient graph is drawn by the Mixed-Model algorithm [39]. To summarize, this algorithm has two steps :

• The first step builds an ordered partition of the set of nodes. This partition is called shelling ordering. The principle is to remove successively nodes that are on the external face of the graph.

• The second one is the "recomposition" of the graph according to the shelling ordering. To guarantee that there is neither edge-edge crossing nor node-edge overlapping, the ordering is traversed in reverse order.

As described in the background section, if a vertex is in a pathway, it has to be drawn close to the other vertices of the pathway. Taking into account such a constraint in the Mixed-Model algorithm can be done during the decomposition phase. Let *SO *= {*V*_1_, *V*_2_, ..., *V*_*r*_} be the shelling ordering. When a vertex *n *is added to a set *V*_*i*_, 1 ≤ i < r, we add in priority vertices which have a constraint with *n *into the next *V*_*j*_, *j *> *i*. Those nodes will be more likely to be drawn next to each other.

The last step of our drawing algorithm is to draw edges removed during the planarization step. These edges are routed on the external face, using an orthogonal drawing with three bends per edge. Figure 4 shows the drawing obtained by our algorithm on the metabolic network of *E. coli*. This is an organism which has been widely studied, its metabolism is composed of 198 pathways, 1140 substrates and reactions (*i.e*. nodes) and 1321 links (*i.e*. edges) between them.

#### Parameter: focus pathways

The algorithm allows to focus on several pathways, *i.e*. one can choose pathways to be entirely clustered. Users constrain the independent set algorithm by giving an ordered list of pathways that are clustered if possible. Indeed, such a list may not be represented by an independent set in the dependence graph (*i.e*. one or more nodes are shared by pathways of the list). In this case, the order of the list gives the priority associated to each pathway and helps to extract an independent set of pathways from the list. Nodes representing those pathways and their neighbors are removed from the dependence graph. An independent set is then computed in the resulting dependence graph. The final independent set is obtained by adding this independent set and those computed in the list.

## Results

### Data

To test and validate the algorithm, we used data from the version 10.0 of the EcoCyc database. We developed perl scripts using the pathway tools software [42-44] to obtain information on the reactions, compounds and metabolic pathways involved in the metabolism of the *K12 *strain of *Escherichia coli*. We chose this organism because it is perhaps the most curated one and we thus avoid most of the data artifacts caused by automatic reconstructions of metabolism.

Several filters are applied on the original data to build our test data. The first one is to withdraw reactions involving large molecules such as proteins. Next, we remove reactions that are involved in no identified metabolic pathway. The last filter has for objective to avoid ubiquitous compounds. Indeed, co-factors such as ATP and NADH participate in many reactions and form hubs in the network which lead to a very fuzzy drawing. One traditional way around this problem is to eliminate the most connected compounds but this implies that metabolic pathways that have these compounds as final products or as precursors become meaningless. We therefore prefer another solution which consists in eliminating the connection between a compound and a reaction if the compound is annotated in EcoCyc as "secondary" in each metabolic pathway that contains the reaction. A compound is defined as "primary" in a BioCyc metabolic pathway when it is a direct chemical intermediate between the start substrate(s) and the end product(s) and is defined as "secondary" when it is a sub-product or a secondary substrates (e.g cofactors) of the metabolic pathway.

It is important to note that this filter leads to a clearer drawing but any kind of compound filter could be applied. In the same way, the classification of the reactions in the EcoCyc-defined metabolic pathways was an easy way to test our algorithm but other classifications could be used, for instance a decomposition into elementary modes [45] or extreme pathways [46]. A metabolic pathway, as defined in BioCyc, can be either a linear chain of reactions, a branched pathway, a cycle: this topological diversity is interesting for testing our drawing algorithm.

The data is stored in a SBML file [47] and computed by MetaViz. The information about the belonging of each reaction is directly included in the SMBL file as shown below in the entry of one reaction which belongs to three different metabolic pathways:

...

<reaction id="DIHYDROFOLATEREDUCT__45__RXN" name="DIHYDROFOLATEREDUCT-RXN" reversible="true">

   <notes>

      <html:p>SUBSYSTEM: tetrahydrofolate biosynthesis</html:p>

      <html:p>SUBSYSTEM: superpathway of chorismate</html:p>

      <html:p>SUBSYSTEM: formylTHF biosynthesis I</html:p>

   </notes>

   <listOfReactants>

      <speciesReference species="THF" stoichiometry="1"/>

   </listOfReactants>

   <listOfProducts>

      <speciesReference species="DIHYDROFOLATE" stoichiometry="1"/>

   </listOfProducts>

</reaction>

...

After the filtering, the SBML file contains :

• 553 compounds and 597 reactions (the nodes of the network represented in Metaviz)

• 198 metabolic pathways of which 30 are superpathways, *i.e*. pathways which contain other pathways.

### Validation

The protocol we adopted for the validation is the following: we systematically compared the behavior of MetaViz to Cytoscape and to the Pathway Tools cellular overview diagram whenever possible. This comparison was carried out for the following tasks:

• Visualization of the whole network;

• Visualization of individual metabolic pathways;

• Visualization of a metabolic pathway in its context.

#### Visualization of the whole network

**Figure 5:**
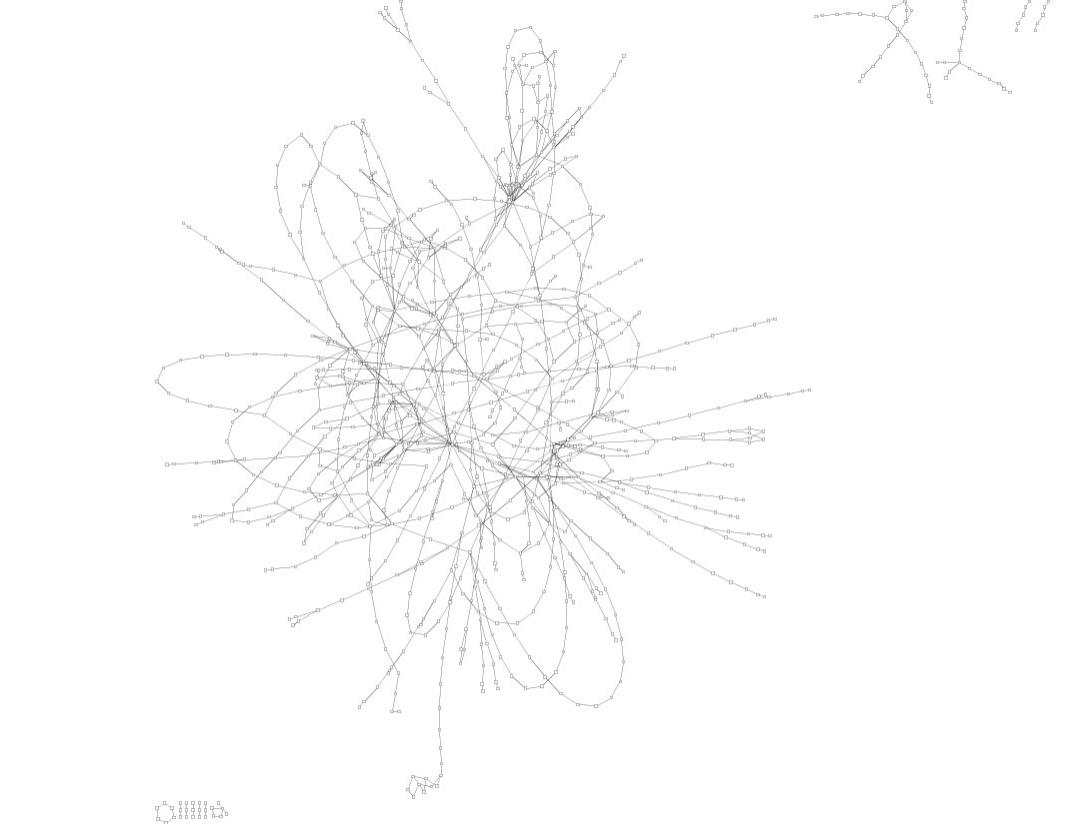
Whole metabolic network of E. coli drawn by Cytoscape.

Figure 4 shows the whole metabolic network computed by MetaViz from the data described in the previous section. Unlike the drawing obtained by Cytoscape [33] with the same data (Figure 5), the metabolic network is organized into metanodes in MetaViz. The purple metanodes indicate the metabolic pathways selected during the clustering step and which are therefore drawn well (nodes of the pathways are close to each other). These metabolic pathways form the backbone of the drawing, which can be changed by choosing to draw well other metabolic pathways.

**Figure 6:**
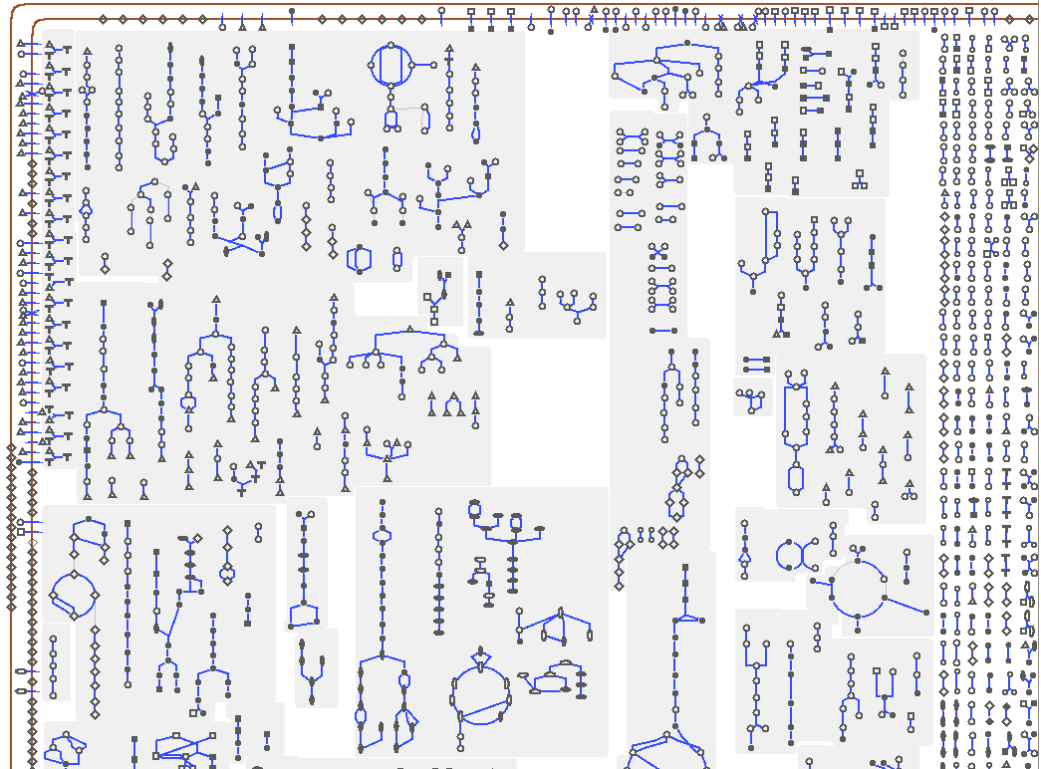
Whole metabolic network of E. coli drawn by the Pathway Tools cellular overview diagram.

The drawing obtained by the Pathway Tools cellular overview diagram (Figure 6) with the same data represents all metabolic pathways but in this case, the layout is fixed. Moreover, it is not possible to zoom further into the drawing.

**Figure 7:**
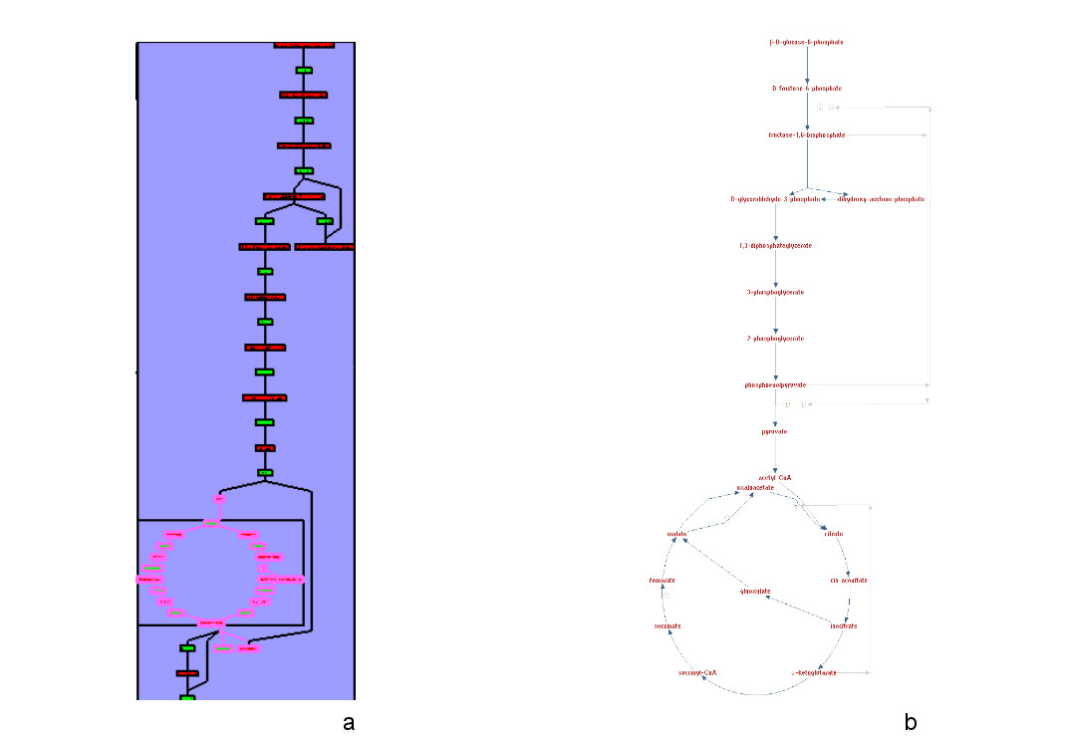
**The superpathway of glycolysis, pyruvate dehydrogenase, TCA, and glyoxylate bypass**. (a) In MetaViz. The nodes corresponding to the TCA cycle are surrounded in pink. (b) In BioCyc.

Unlike the Pathway Tools cellular overview diagram, MetaViz enables to see a metabolic pathway in its context, keeping the same layout. For instance, Figure 7a is merely a zoom of Figure 4.

#### Drawing of the TCA cycle

We do not compare the results with Cytoscape of which the purpose is not to draw metabolic pathways but only to draw a whole network.

In the data from BioCyc, the TCA cycle is included in the super pathway of "glycolysis, pyruvate dehydrogenase, TCA, and glyoxylate bypass". Because of its great number of nodes, this pathway was chosen by the algorithm to be particularly well drawn: all the nodes (compounds and reactions) involved in this super pathway are grouped together into a same metanode (Figure 7a). The drawing obtained by MetaViz is very similar to the one obtained by the pathway viewer of BioCyc (Figure 7c). The differences between the two drawings are mostly due to the differences in the types of graph used to model the network: a simple graph in the case of BioCyc, and a bipartite graph in the case of MetaViz.

#### Drawing of the valine biosynthesis pathway

This pathway is a four-step chain which starts with pyruvate and ends with L-valine.

**Figure 8:**
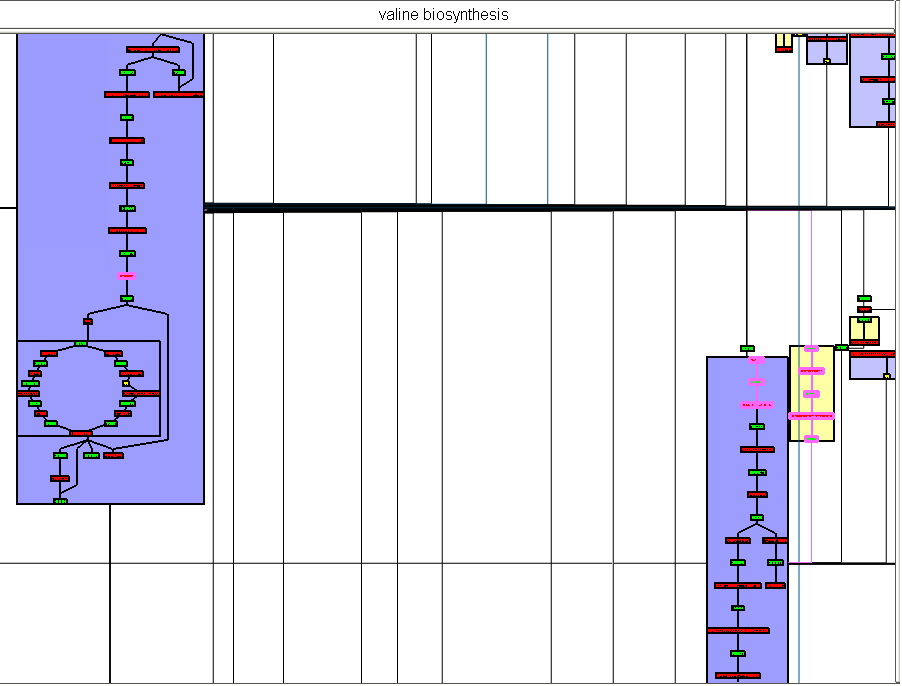
**Valine Biosynthesis pathway in MetaViz, without choosing the metabolic pathway to be well drawn**. The corresponding nodes are surrounded in pink and we can see that they are shared by 3 metanodes.

We present here two cases: 1. the clustering is not guided and 2. the clustering is guided. If the clustering is not guided, this pathway is not selected to be drawn well and is actually split into three parts: one node is drawn in the superpathway of the TCA cycle and glycolysis (because they share the pyruvate), one node corresponds to the superpathway of pantothenate and coenzyme A biosynthesis (because they share L-valine, alpha-keto isovalerate and the 2.6.1.42 reaction) and the third node corresponds to the other reactions (Figure 8).

**Figure 9:**
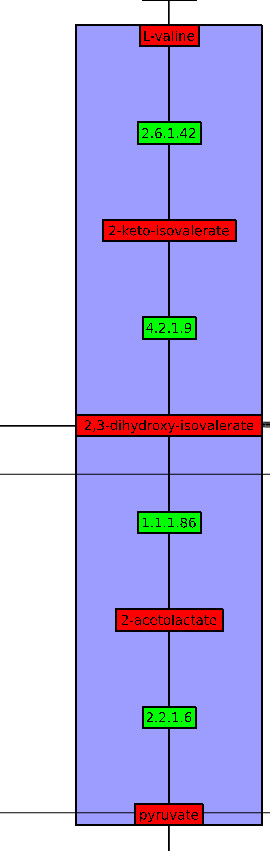
Valine Biosynthesis pathway in MetaViz, after choosing this metabolic pathway to be drawn well.

**Figure 10:**
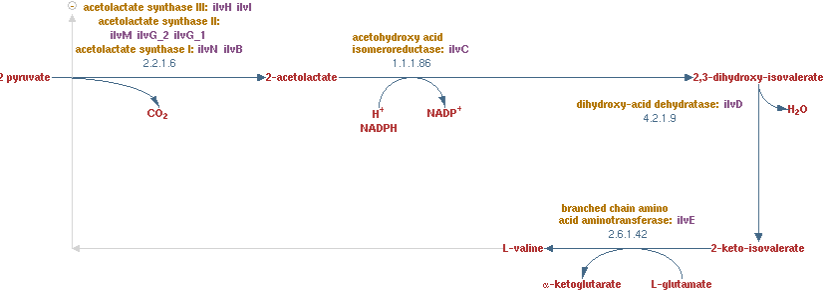
Valine Biosynthesis pathway in BioCyc.

This metabolic pathway has not been efficiently drawn because some of its elements belong to larger metabolic pathways. Nevertheless, we do not see such a representation as a negative result but instead consider the division of this metabolic pathway into several parts as interesting. Indeed, it means that this metabolic pathway shares several elements with others, showing the interdependence between the pathways. Otherwise, if the clustering is guided and valine biosynthesis is chosen as a focus pathway, MetaViz efficiently represents it (Figure 9). Obviously, this choice leads to the disconnection of the metabolic pathways sharing the same nodes. As mentioned above, we can see here one of the main interests of MetaViz: it is possible to change the backbone of the drawing to center it on specific metabolic pathways. If we compare this drawing with the one obtained by the pathway viewer of BioCyc (Figure 10), we observe that the order of the nodes is reversed. That is pyruvate is on the left of BioCyc drawing while it is at the bottom of the MetaViz one. Hence pyruvate appears as the input of the pathway. But in BioCyc SBML description these reactions are annotated as reversible. So it is not, in that case, possible to automatically identified pyruvate as the input of the pathway.

#### Visualization of a metabolic pathway in its context

MetaViz represents explicitly the links between metabolic pathways. These links are ignored when metabolic pathways are separately drawn (as in BioCyc) or when no information about the belonging of the nodes to a metabolic pathway is displayed (as in Cytoscape). The Pathway Tools Cellular Overview diagram proposes to optionally draw these links in superposition to the main drawing. The limit of this approach is that, since these links are not incorporated in the original layout, the final drawing may become very dense and hard to read.

**Figure 11:**
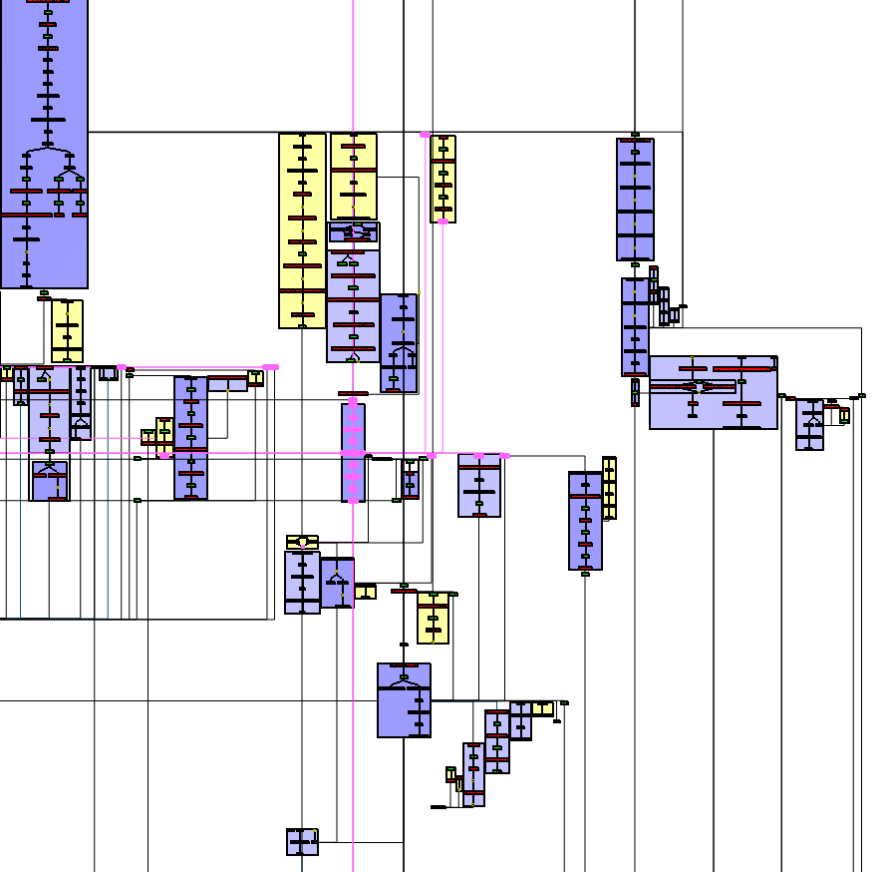
Drawing of the nodes (colored in pink) directly connected to the Valine Biosynthesis Pathway (in the center of the figure).

It is possible with MetaViz to highlight the nodes that are neighbors of a selected node. Figure 11 shows the direct neighbors (colored in pink) of the valine biosynthesis pathway. One can then more easily follow each edge to see to which nodes in the network this metabolic pathway is connected.

**Figure 12:**
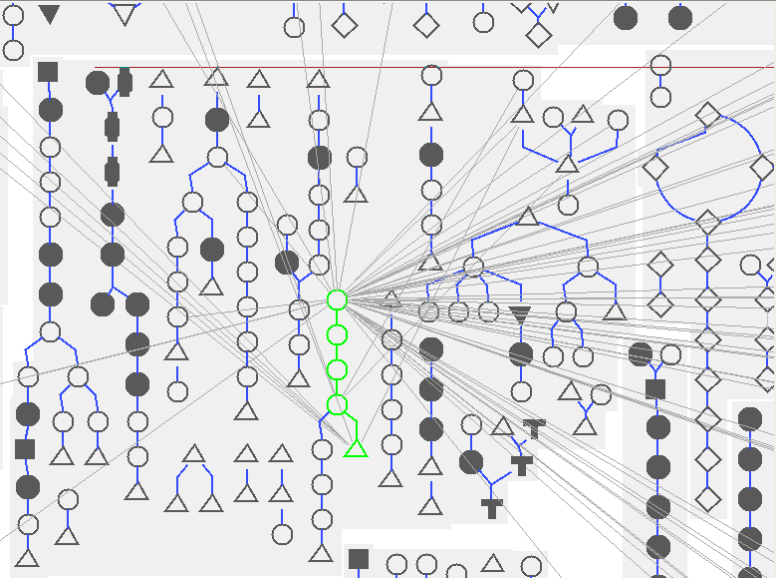
Connections from the valine biosyntheisis pathway in the Pathway Tools cellular overview diagram.

Figure 12 shows the connections from the valine biosynthesis pathway computed in the Pathway Tools cellular diagram overview. However, because nodes are duplicated and the layout is fixed, a lot of edges are displayed and it is difficult to follow one edge.

## Conclusion

In this paper, we present an algorithm to compute the representation of a metabolic network. This method addresses a challenging problem which consists in representing simultaneously the topology and the metabolic pathway information. Indeed, metabolic pathways often share metabolites and reactions, thus to represent them in a single view, previous approaches duplicated these shared elements. However, duplication produces drawings where the depicted connectivity does not fit the real topology of the network. To overcome the problem of shared nodes, we propose a clustering step based both on topology and a metabolic pathway decomposition. During this step, we deal with pathway overlapping by detecting a largest set of independent pathways and sub-pathways. The resulting graph clustering shows the overall organization of the pathways. To follow common drawing conventions, it is drawn using a planar graph drawing algorithm. Finally, each pathway or sub-pathway is drawn using specific drawing algorithms (hierarchical and circular ones). In our collaboration with physiologists, we noticed that they often consider some pathways as being central in their global studies. To respect their habits, the physiologists can provide a set of focus pathways that will be considered as a parameter of the clustering step. Thus our algorithm will generate a drawing where these pathways are entirely and carefully drawn.

This global representation allows the visualization of processes that span over different metabolic pathways. For instance, this approach was successfully used to highlight metabolic processes, especially those traversing different metabolic pathways.

One of the future directions we would like to consider concerns the improvement of the global aspect of our drawing. The drawing conventions that we identified for metabolism are mostly local (emphasizing cycles and reaction cascades). Following them does not ensure to have a global picture that will look like the Boehringer map [23] which may be closer to what biochemists are used to. Indeed, the global picture that we obtain with our method can be puzzling at first glance, and it is only when navigating in the drawing that the user will find more familiar patterns. We believe that we can improve the aspect of the global drawing in considering alternative ways of drawing the quotient graph.

In this paper, we focused on the drawing part of metabolic network visualization. As it was mentioned, drawings are used as a background for high throughput data visualization. Since this algorithm is already implemented in a graph drawing software [38], we plan to develop an input module for omic data. Another issue will be to add more relational information such as signaling processes. We plan to use the third dimension to incorporate the additional edges.

## Availability and requirements

Project name: MetaViz

Project home page: 

Operating system(s): Currently Linux and Windows. Mac OSX ports is possible.

Programming language: C++

Other requirements: Tulip [38], Qt from Trolltech.

License: GPL

## Authors' contributions

FJ initiated this work. RB, VL, LC, DA, MS and FJ defined metabolic network drawing constraints. RB, DA, and FJ established the translation of these constraints into graph drawing ones. RB and DA designed the drawing algorithm. RB and PM implemented the algorithm. LC build the datasets from EcoCyc. VL, LC and MS performed the tests and result analysis. All authors participated in manuscript preparation. All authors have read and approved the final manuscript.
